# In sync with oneself: spontaneous intrapersonal coordination and the effect of cognitive load

**DOI:** 10.3389/fnhum.2025.1457007

**Published:** 2025-03-24

**Authors:** Ramkumar Jagadeesan, Jessica A. Grahn

**Affiliations:** ^1^Department of Psychology, Western University, London, ON, Canada; ^2^Western Centre for Brain and Mind, Western University, London, ON, Canada

**Keywords:** coordination, synchronization, intrapersonal, spontaneous, coupling, attention, cognitive load, interpersonal

## Abstract

Spontaneous intrapersonal coordination is the unintentional coordination of periodic behaviors within an individual. Spontaneous interlimb coordination involving finger-, arm-, foot-, leg- and orofacial muscle movements may be weaker between finger-tapping and walking than between finger-tapping and vocalizing. This could be due to the additional attentional cost of walking, which may be more complex than other periodic movements. Here we compared the coordination stability of simultaneous finger-tapping and walking against simultaneous finger-tapping and repetitive vocalization. We also tested the coordination stability of tapping-walking and tapping-vocalizing under additional cognitive load imposed through concurrent cognitive tasks. Two experiments conceptually replicated spontaneous intrapersonal coordination between the pairs of periodic tasks as well as the effect of concurrent cognitive tasks on coordination stability. To assess coordination, we compared the phase coherence of two periodic tasks, tapping with walking (Experiment 1) or tapping with vocalization (Experiment 2), when produced separately (single task) versus simultaneously (dual task). In the first experiment, participants regularly tapped a microphone while walking, either with no concurrent cognitive task or with concurrent backward counting. In the second experiment, participants tapped while repeating the word “tick,” again either with no concurrent cognitive task, or with concurrent visual pattern-matching. Higher spontaneous intrapersonal coordination was evident between periodic tasks when performed simultaneously compared to separately, and lower task coordination stability was evident with a concurrent cognitive task compared to without. These results were in line with past findings. Coordination stability between tapping and walking was lower than that between tapping and ticking overall. This finding supports the categorization of walking as a more complex cognitive task compared to other periodic tasks, as the additional attentional load involved in walking could have resulted in lower coordination stability between tapping and walking. Spontaneous intrapersonal coordination appears sensitive to the attentional costs of performing periodic activities and achieving / maintaining coordination between them.

## 1 Introduction

Coordinated movement is such an integral component of human nature that it often goes unnoticed. For example, imagine an audience, watching musicians and dancers perform, and grooving along with the artists by tapping their feet, snapping their fingers, clapping their hands, and bobbing their heads. These periodic behaviors often interact and influence each other unintentionally. The unintentional temporal coordination that emerges spontaneously between periodic movements may occur intrapersonally, as in the case of interlimb coordination during bimanual finger-tapping (Kelso, 1984; Lorås et al., 2019). Coordination may also occur interpersonally, as in the case of spontaneous synchronization of footsteps (while walking together) observed in dyads (Zivotofsky and Hausdorff, 2007) and pedestrian crowds (Fujino et al., 1993; Ma et al., 2021).

Although “coordination,” especially interpersonal, is often referred to as “synchronization” in the literature, coordinated periodic behaviors seldom happen strictly at the “same time or rate” as per the lexical definition of synchronization. This is because synchronization between individuals is not perfect, because there is variability. For example, although stride time variability in healthy young adults is under 3% in general (Beauchet et al., 2009), it is not zero. Synchronization therefore requires individuals to coordinate their individual rates to adopt either the same rate or a related rate (harmonic or subharmonic) such that the relative asynchronies between corresponding repetitions of the behavior (e.g., step times) tend toward a constant value. This applies not only to synchronization involving walking, but also to other periodic tasks such as finger-tapping, where inter-tap interval varies with every tap (Yamada, 1995). So, two partners performing a periodic behavior together will likely have slightly different rates, say, f_1_ and f_2_, respectively instead of a common rate (F), and also, the exact times of their corresponding repetitions, say, t_1_ and t_2_, respectively, will likely be slightly different as well, such that t_1_ - t_2_ ≠ 0. Under synchronization, t_1_ - t_2_ will tend toward a constant value. Fraisse, in the context of sensorimotor synchronization of finger taps to periodic auditory stimuli, made this observation as early as 1966; he termed it as quasi-simultaneity and categorized this form of coordination as a viable mode of synchronization (Fraisse, 1966; Fraisse and Repp, 2012). Definitions of interpersonal synchronization over the years echo Fraisse’s categorization: “Rhythmic coordination of perception and action” (Repp, 2005, p. 969), “coordination of rhythmic movement with an external rhythm” (Repp and Su, 2013, p. 403), and “temporal coordination between humans” (Tranchant et al., 2022), are some of the definitions of synchronization found in the literature as on date. We make this point about synchronization and coordination at the outset to clarify that, contrary to Fraisse’s categorization of coordination as a viable mode of synchronization, coordination subsumes synchronization: Synchronization, as predicted by the coordination pattern dynamics model, is the most stable pattern of coordination (Haken et al., 1985). This clarification is to avoid confusion due to interchangeable usage of the terms “synchronization” and “coordination” going forward.

The stability of coordination between interacting periodicities depends upon their coupling strength, which refers to the intensity of the influence between two systems. This influence can be either unidirectional or bidirectional (Haken et al., 1985; Boccaletti et al., 2002). Stronger coupling increases the likelihood of coordination between behaviors. Interpersonal coupling is typically achieved through exchange of sensory feedback – visual, auditory, or tactile. Exchange of tactile feedback by holding hands has proved to be the most effective in eliciting spontaneous synchronization of gait (Zivotofsky and Hausdorff, 2007; Zivotofsky et al., 2012, 2018; Sylos-Labini et al., 2018). Although not as consistent as their tactile counterpart, visual and auditory feedback have, in some cases, been effective in triggering an increase in spontaneous interpersonal synchronization of various activities, including swinging a handheld pendulum, swaying in a rocking chair, walking, and running (Schmidt and O’Brien, 1997, Richardson et al., 2007; Oullier et al., 2008; Lopresti-Goodman et al., 2008; Harrison and Richardson, 2009; Zivotofsky et al., 2012). Even in naturalistic settings, spontaneous synchronization seems to follow the natural exchange of sensory feedback between interacting entities. Audiences spontaneously synchronize their applause – what begins as random, incoherent clapping becomes synchronized (Néda et al., 2000a,2000b,^2003^). Also, humans synchronize their footsteps spontaneously when walking with others; such observations have been reported in dyads (Zivotofsky and Hausdorff, 2007), as well as in crowds of pedestrians (Fujino et al., 1993; Ma et al., 2021). As strong as spontaneous interpersonal coupling is, enough to trigger synchronization, it is not as strong as spontaneous intrapersonal coupling and the resultant coordination (Schmidt et al., 1998). Intrapersonal coordination occurs spontaneously in bimanual tapping (Kelso, 1984; Loras et al., 2019), between arm and leg movements (Sakamoto et al., 2007), as well as between limb movements and orofacial muscle movements, such as walking and chewing gum (Samulski et al., 2019). When performed concurrently, finger-tapping and speaking influence each other in terms of rates, variabilities as well as stress patterns (Hiscock et al., 1985, Smith et al., 1986; Parrell et al., 2011).

In a study by Qi et al. (2019) participants aged 20–30 years performed finger-tapping at a given inter-tap interval of 375 ms with concurrent foot movements at a given inter-(heel)-strike interval of 600 ms; the foot movements were alternative bilateral heel tapping from a sitting position, and unilateral heel tapping with the leg ipsilateral to the tapping finger from a sitting position. Each participant also performed the finger-tapping task (at 375 ms inter-tap interval) with concurrent walking, both at given pace (at 400, 600, and 800 ms inter-step intervals) as well as at preferred pace (self-paced). Despite the given inter-repetition intervals for finger-tapping and heel-striking being unrelated (by design), spontaneous interlimb coordination of finger-tapping was significant with all the concurrent foot movements except, however, with given-paced walking and self-paced walking. Researchers concluded that tapping and walking could be done with “independent rhythms”. In general, weaker coordination could be indicative of higher attentional cost of intentionally maintaining the coordination (Zanone et al., 2001; Temprado et al., 1999; Pellecchia et al., 2005). In this study, the weaker coordination between finger-tapping and walking (compared to heel tapping) could have been due to higher attentional cost involved in walking. Evidence suggests that walking, compared to other periodic tasks like finger-tapping, could be a more complex cognitive activity (Sheridan and Hausdorff, 2007; Hausdorff et al., 2005). Given that, the attentional cost required to intentionally maintain the coordination of finger-tapping with walking would be more compared to that of finger-tapping with other periodic tasks, explaining the finding by Qi et al. (2019).

The above explanation could be further tested by comparing the stability of coordination of finger-tapping and walking against that of finger-tapping and other periodic tasks. For such a comparison, repetitive vocalization could be a suitable periodic task to be paired with finger-tapping as concurrent finger-tapping and speaking influence each other in terms of rates, variabilities as well as stress patterns (Hiscock et al., 1985, Smith et al., 1986; Parrell et al., 2011). Furthermore, stability of bimanual coordination decreases with concurrent cognitive tasks such as reaction time task (Temprado et al., 1999) and backward counting task (Pellecchia et al., 2005). This is understandable given how the variability of finger-tapping (as a single task) increases with concurrent n-back task (Kirchner, 1958) or mental arithmetic tasks (Irie et al., 2022; Bååth et al., 2016). In that vein, given how concurrent backward counting affects gait speed and variability (Li et al., 2014; Beauchet et al., 2005), it would be understandable if the stability of coordination involving walking decreased with concurrent backward counting. Therefore, put together, we could expect the stability of coordination between tapping and walking to decrease with concurrent backward counting. Also, finger-tapping and vocalizing simple repeated sequences interfered with performance in the concurrent Multiple Object Tracking (MOT) task (Trick et al., 2006), suggesting that all three tasks shared attentional resources. Therefore, we could expect the stability of coordination between tapping and repetitive vocalization to decrease with a concurrent visuospatial task. Further, findings to date are unclear as to whether the concurrent attentional load could be altered by varying task difficulty. For example, backward counting in 3′s versus 7′s has been found to alter concurrent attentional load in some cases (Kroll and Kellicutt, 1972) while not in others (Houghton et al., 2003). Given that, it is worth testing if varying task difficulty varies the effect of the concurrent cognitive task on coordination stability.

In the current study, we compared the stability of spontaneous (unintentional) coordination of repetitive finger-tapping with walking as well as with repetitive vocalization, where all the periodic behaviors were at preferred rates. Here, any difference between tapping-walking and tapping-vocalization in terms of coordination stability could partially be due to the difference in attentional costs of walking and repetitive vocalization. To isolate that part, the attentional costs incurred through other factors had to be minimized. To that effect, first, unintentional coordination was compared instead of intentional coordination for the dual tasks as the former has been found to incur less attentional cost (Aubin et al., 2021); second, coordination at preferred rates was compared instead of the same at given rates as the attentional cost is at its minimum when coordination pattern is at preferred frequency (Zanone et al., 2001). In the current study, we also compared the effect of backward counting across difficulty levels on spontaneous tapping-walking coordination, as well as the effect of matching visual-patterns across difficulty levels on spontaneous tapping-vocalization coordination.

We conducted two conceptual replication experiments. In each experiment, we tested the stability of spontaneous (unintentional) intrapersonal coordination between the periodic behaviors at preferred rates, with and without concurrent cognitive task. The research question was, with no concurrent cognitive task, would the stability of coordination of finger-tapping be lower with walking than with repetitive vocalization? The evidence showing walking as a more complex cognitive task compared to other periodic tasks was not enough to assume that repetitive vocalization was one of the other periodic tasks, rendering any hypotheses in response to the research question not justified enough. We therefore made *post hoc* comparisons of the two periodic task pairs (tapping-walking and tapping-vocalization) in terms of their coordination stability when no concurrent cognitive task was performed. As the cognitive tasks were different across the two experiments for reasons discussed above, such a comparison was not meaningful when the concurrent cognitive task was performed.

## 2 Materials and methods

### 2.1 Participants

Twenty-four participants (mean age = 22.58 years; range = 18–33; SD = 5.5; 10 males and 14 females; all right-handed) were recruited for the study comprising 2 experiments. Each participant completed both experiments. Age, gender and dominant hand were self-reported. Fourteen participants were recruited from the Psychology research participation pool at Western University and received 1 course credit compensation. The remaining 10 participants were recruited from students and the general public and compensated $10 for the 1-h study. The study was approved by the Non-Medical Research Ethics Board at Western University.

### 2.2 Design

**FIGURE 1:**
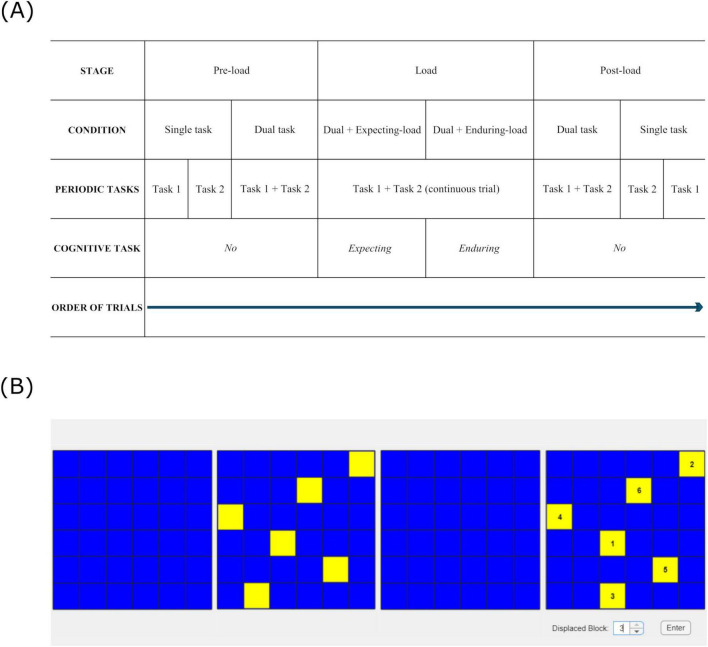
**(A)** Study design for both experiments – (6 conditions across 3 stages), **(B)** Visual pattern-matching task: Matrices show the timed sequence (from left to right) in which a pattern was presented for matching over the course of a load stage trial. The task was to be completed while tapping and ticking simultaneously for the entire trial: (far left) waiting for the pattern to appear (15 s); (second from left) encoding the pattern (6.5 s); (second from right) retaining the pattern that disappeared (3.5 s); (far right) matching the pattern that reappeared with the encoded one for identifying any difference (∼ 5 s).

The study comprised two experiments, completed in a single session lasting 1 h. The order of completion of the experiments was counterbalanced across participants. The study design (see Figure 1A) was common to both experiments.

Each experiment had a different pair of periodic tasks (performed separately as single tasks, and simultaneously as dual task), and a different cognitive task with multiple difficulty levels (performed concurrently with dual task). Experiment 1 had repetitive finger-tapping and walking as periodic tasks, and backward counting as the concurrent cognitive task with two difficulty levels (counting backward in 3′s and 7′s). Experiment 2 had repetitive finger-tapping and repetitive vocalization of the word “tick” (ticking) as periodic tasks, and visual pattern-matching as the concurrent cognitive task with six difficulty levels (matching patterns comprised of 4–9 blocks). The word “tick” was selected for repetitive vocalization as piloting showed it was easy to extract durations between successive peak intensities in the audio waveform if a short vowel with a glottal stop was used to reduce vowel duration variability. Backward counting was not used in Experiment 2 as it was incompatible with vocalizing “tick”. As matrix patterns are not easily verbalizable (Della Sala et al., 1999), visual pattern-matching task was used instead. The task involved determining whether sequentially presented patterns on two adjacent 6x6 matrices were the same or different. Six levels of cognitive load were induced by manipulating the number of blocks in the patterns, from 4 to 9. The task was designed based on a 2005 study where simultaneous presentation of patterns were used in a matching task, where subjects were allowed 1 s to encode each block in the pattern: for example, 5 s would be allowed to encode a 5-block pattern (Lecerf and de Ribaupierre, 2005). This was in line with the common block-tapping rate of 1 s per block, used widely in the administration of the Corsi Block Test (Arce and McMullen, 2021; Corsi, 1972). Therefore, we allotted 6.5 s on average to encode each pattern with 4 to 9 blocks.

Each experiment had 3 cognitive load stages: pre-load, load, and post-load. During the pre-load stage, the two periodic tasks were performed separately (single task) as well as simultaneously (dual task), always in that order. During the load stage, we assessed whether dual task performance was affected by the anticipation of the concurrent cognitive task (“expecting-load” condition); we also assessed dual task performance with concurrent cognitive task (“enduring-load” condition). Load stage trials ran the expecting-load condition for the first half, and the enduring-load condition for the second half. Expecting-load was treated as a separate condition as the anticipation of dealing with the imminent demands of cognitive functioning could worsen working memory performance (Hyun et al., 2019). During the post-load stage, we tested for any persisting effects of the load stage (e.g., any change in coordination stability that may have occurred then). Here the order of dual task and single task conditions was reversed compared to the pre-load stage. Also, the order of completion of the periodic tasks in the single task condition was reversed across pre-load and post-load stages. The specific periodic task (e.g., tapping or walking) that was assigned to be “Task 1” and “Task 2” was counterbalanced across participants.

### 2.3 Materials

For Experiment 1, walking data was captured using the Zeno Walkway gait mat and ProtoKinetics Movement Analysis Software (PKMAS); tapping was audio recorded in version 3.3.3 of Audacity^®^, a free software distributed under the terms of the GNU General Public License.^1^ For all audio recordings, Fifine Technology Bluetooth receivers and wireless microphones, Focusrite Scarlett 2i2 audio interface, and Windows laptop were used. Sound intensity data were extracted from audio recordings using version 6.2.14 of Praat, a speech analysis software in phonetics (Boersma and Weenink, 1992–2022). Data were sorted in Microsoft Excel (2016). Metrics were calculated using code written for the study in MATLAB (R2022a). Data analyses were done in Jamovi (2.3.21). Graphing was done in JASP (0.18.3).

### 2.4 Procedure

The participants were seated at a table on which the laptop running Audacity was placed. The Bluetooth receivers were plugged into the Focusrite audio interface, one on each channel. There were two wireless microphones; one of them was used as a hand mic for tapping (Experiments 1 and 2), and the other one was used on the stand for ticking (Experiment 2). Participants were instructed to use their non-dominant hand for holding the hand mic, and the index finger of their dominant hand for tapping on the mic. The dominant and non-dominant hands were self-reported. The hand mic was paired to the receiver on line 1 of the audio interface, and the stand mic was paired to the receiver on line 2; this was to make sure the signal from the hand mic was always recorded onto the left stereo channel, and that from the stand mic was always recorded onto the right. Audio input levels were checked and optimized for each participant before the start of the trials, so that the signals were neither too low nor too high. In Experiment 1, at the start of each trial involving the simultaneous performance of the periodic tasks, the hand mic was tapped gently on the gait mat by the experimenter to create two events of reference at the same timepoint, one on the gait mat recording of the walking task, and the other on the audio recording of the tapping; this was done to create a synchronization trigger between the audio and gait data, for temporally aligning tapping and walking responses. Signed informed consent was obtained from each participant.

#### 2.4.1 Experiment 1—walking and tapping

Participants practiced counting backward before completing the tasks in the following order.

Pre-load stage

Single task condition

Tapping: Participants were instructed to tap on the hand mic repetitively at whatever rate felt natural. For each trial, they were to start and stop tapping as prompted by the experimenter. They completed 2 trials, lasting 20 s each, with a 20 s break.

Walking: The participants were instructed to start walking at whatever rate felt natural from just behind a tape line on the floor, marking 1.78 m from the edge of a 4.88-m Zeno pressure-sensor gait mat, and continue walking across the mat to its other side; they were instructed to maintain their stride as they walked off the mat a further 1.78 m on the other side, marked by another tape on the floor, before making a wide U-turn to walk back onto the mat for the next lap. For each trial, they were to start and stop walking as prompted by the experimenter. They completed 2 trials of 4 laps each, with a 20 s break.

In this stage, all participants completed tapping first, followed by walking.

Dual task condition

The participants were instructed to tap and walk simultaneously at whatever rates felt natural. For each trial, they were instructed to start and stop both tasks at the same time, as prompted by the experimenter. They completed 2 trials of 4 laps each, with a 20 s break.

Load stage

During the first half of each trial, from lap 1 to 3 (expecting-load condition), participants were instructed to tap and walk simultaneously at whatever rates felt natural, while waiting to begin counting backward from lap 4 when the enduring-load condition would commence (without a break). During the second half, from lap 4 to 6 (enduring-load condition), they were instructed to continue tapping and walking simultaneously at whatever rates felt natural, while counting backward from a 3-digit number (between 600 and 999) using a negative counter (3 or 7, representing cognitive load levels 1 and 2, respectively); the number and the counter were given at the start of the trial. They completed 4 trials, 2 at load level 1 followed by 2 at level 2.

Each load stage trial lasted 6 laps instead of 8 for the following reason: During piloting, some participants indicated it was taxing to keep track of which lap they were in during the expecting-load condition, which they needed to do to know when to begin counting backward for the enduring-load condition. To avoid such taxation, if needed, the experimenter visually cued the participants to count backward as they were about to start the enduring-load condition. Such cueing was more seamless at the start of the even-numbered laps, when the participants faced the experimenter, than the odd-numbered ones, when they faced away. This made lap 4 more preferable, instead of lap 5, to begin counting backward; such a preference was therefore accommodated in the trials.

Post-load stage

The dual task condition was performed first with no additional cognitive load, followed by the single task condition. The order of completion of the periodic tasks in the single task condition was reversed compared to the pre-load stage: walking followed by tapping.

#### 2.4.2 Experiment 2—tapping and ticking

A customized application developed using the MATLAB App Designer was used to administer the tasks and record task performance. The primary purpose of using the application was to administer the trials in the load stage conditions, where simultaneous presentation of the visual pattern-matching task and recording audio of taps and ticks was needed. Subsequently, for consistency of task administration interface across stages, we used the application for administering the pre-load and post-load stage trials as well. Before the trials, the participants were briefed about the visual pattern-matching task, after which they practiced the task through the “demo” version on the application. Participants completed the tasks in the following order.

Pre-load stage

Single task condition

In this stage, participants completed tapping first, followed by ticking.

Tapping: Participants were instructed to tap on the hand mic repetitively at whatever rate felt natural. For each trial, they were to start and stop tapping as prompted by the MATLAB application. They completed 2 trials, lasting 15 s each, with a 15-s break.

Ticking: The participants were instructed to repeat the word “tick” into the stand mic at whatever rate felt natural. For each trial, they were to start and stop ticking as prompted by the MATLAB application. They completed 2 trials, lasting 15 s each, with a 15-s break.

Dual task condition – The participants were instructed to tap and repeat the word “tick” simultaneously at whatever rates felt natural. For each trial, they were instructed to start and stop both tasks at the same time, as prompted by the MATLAB application. They completed 2 trials of 15 s each, with a 15-s break.

Load stage

During the first half of each trial (expecting-load condition lasting 15 s), the participants waited for a pattern to appear on the computer screen. While waiting, they tapped and ticked simultaneously at whatever rates felt natural. Enduring-load condition commenced after 15 s when a pattern comprised of yellow blocks appeared on a blue 6x6 matrix on the left side of the screen. In this condition, participants continued tapping and ticking simultaneously at whatever rates felt natural. Additionally, they encoded the pattern for 6.5 s, at which point the pattern disappeared. They retained the pattern in memory for the next 3.5 s, at which point the pattern reappeared on a similar matrix on the right side of the screen. The yellow blocks on the pattern that reappeared would be numbered, with one of them possibly displaced. Participants identified the displaced block, if any, by matching the pattern on the screen against the one encoded in memory. They entered the number on the displaced block as the answer, or entered “0” if none was displaced. Matching lasted for ∼5 s (see Figure 1B). Total trial duration was ∼30 s. They completed 12 trials, 2 at each cognitive load level, from 1 to 6 based on the number of blocks in the pattern (4–9, respectively).

Post-load stage

The dual task condition was performed first without additional cognitive load, followed by the single task condition. In the single task condition, ticking was performed first, followed by tapping.

#### 2.4.3 Data extraction

Walking: First contact times of footsteps were extracted for each trial with gait mat movement analysis software (PKMAS), and exported to Excel.

Tapping and Ticking: For Experiment 1, the recorded stereo track of the tapping audio on Audacity was split into left and right mono tracks, and the left mono track (tapping) was exported as a wave file. For Experiment 2, the split gave two mono tracks: left (tapping) and right (ticking). Tracks were imported into Praat. In Praat, sound intensity data were extracted from the intensity listings. For each trial, start and end times, as well as the number of sound events (taps or ticks), were extracted manually from the intensity waveform. The extracted data were imported into Excel, sorted, and imported into the MATLAB program that extracted the timing of each sound intensity peak for each tap or tick in each trial.

(MATLAB function for finding peaks in audio was not reliably accurate in extracting event times, especially for ticking. The customized MATLAB code generated for extracting “tick” times worked best with sound intensity data from audio files readily exportable from Praat. Although this meant more extraction steps, we opted for them for reliable extraction accuracy. For consistency, we followed these steps to extract tap times as well).

#### 2.4.4 Phase coherence

We used phase coherence to measure coordination stability. Phase coherence refers to how aligned two periodic inputs are with each other over time. If the phases are in a fixed relationship with each other, they are said to be fully phase-coherent. When phase-coherent, the oscillators may not necessarily be perfectly synchronized in terms of having the exact same phase (i.e., they could still be offset by a constant phase difference), but their phases exhibit a stable relationship. Phase coherence ranges from 0 to 1, where 0 means no coordination at all and 1 means absolute coordination (synchrony). Standard deviation (SD) of relative phase is the standard measure of coordination stability in the literature. However, phase coherence was chosen as its scaling from 0 to 1 offers a more intuitive interpretation of coordination stability, and it is equally sensitive to the variability of relative phase.

**FIGURE 2:**
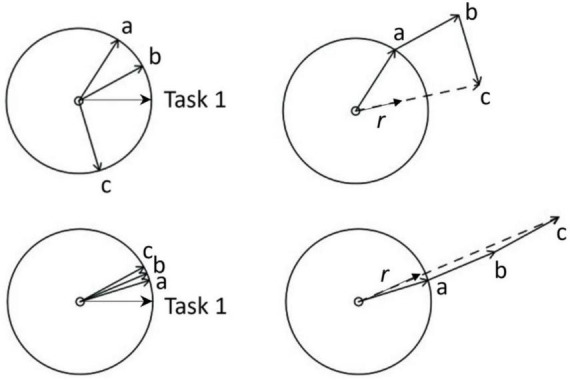
Phase angles [a, b, c] of Task 2 vector relative to Task 1 vector, indicating the positions of one periodic task in the event cycle (Task 2 event in this case – e.g., a footstep) when the other periodic task event occurs (Task 1 event in this case – e.g., a finger tap). Higher phase angle clustering (bottom left compared to top left) of Task 2 relative to Task 1 resulting in greater length (*r*) of the average Task 2 vector (bottom right compared to top right), reflecting higher phase coherence as a measure of higher coordination stability. For example, lower variability of time difference between corresponding events of periodic tasks (e.g., between corresponding footsteps and finger-taps) means lower variability (higher clustering) of relative phase angles, and therefore, higher phase coherence between the tasks.

For each trial, phase coherence was calculated for each periodic task from its relative phase angles (θ_j_) by applying the global order parameter of the Kuramoto model (Acebrón et al., 2005; Kuramoto, 1975). This parameter is a measure of synchronization, quantifying how well the phases of the two task inputs (e.g., taps and ticks) are aligned. As illustrated in Figure 2, each periodic task is a phase vector, and each individual repetition of a periodic task is a cycle. Imagine tapping is task 1 and ticking is task 2. The instant a tap occurs, the task 1 vector is at 0° to the X-axis; also, at that instant, the phase vector representing the ticking task, the task 2 vector, would make a relative phase angle with the task 1 vector. As task 1 is the reference in this illustration (Figure 2), the task 1 vector is fixed at 0°; a, b, c are relative phase angles made by the task 2 vector relative to the task 1 vector, such that, θ_j_ = [a, b, c]. We then applied the global order parameter (to “apply” means to calculate over time) of the Kuramoto model to θ_j_ using Equation 1 below: In this general model of coupled oscillators, θ_j_ is represented as an array of *N* complex numbers e^*i*θ^_*j*_, the average of which represents the average value of the task 2 vector both in terms of length as well as phase angle.


(1) $$r\,e^{i\,\psi }=\frac{1}{N}\,\sum_{j=1}^{N}e^{i\,\theta_{j}}$$

Here, “*r*” is the length of the average vector, ψ is the phase angle of the average vector, and “*i*” is the imaginary unit √−1. The value of “*r*” represents phase coherence (0 ≤ *r* ≤ 1 for an array of unit vectors), indicating the degree to which the relative phase angles (θ_j_) are clustered. As clustering increases, the length of the average vector increases, indicating an increase in phase coherence, and thereby, coordination stability. Phase coherence *r* was determined according to Equation 2 below (Acebrón et al., 2005; Kuramoto, 1975).


(2) $$r=\left|\frac{1}{N}\,\sum_{j=1}^{N}e^{i\,\theta_{j}}\right|$$

**FIGURE 3:**
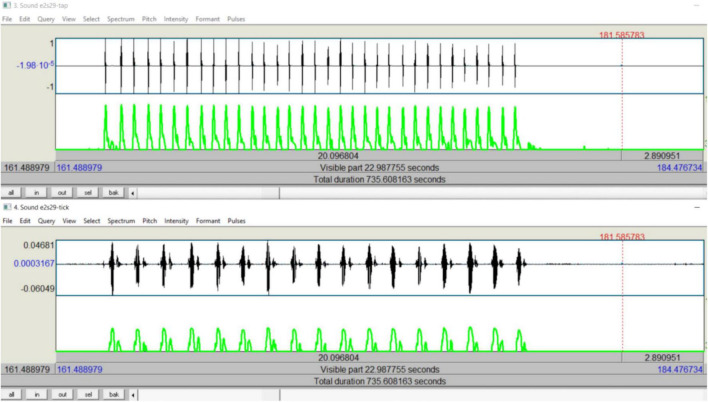
Sound intensity representations of a dual-task trial: screenshot showing temporal alignment between tapping and ticking performed simultaneously.

For each trial, for each task, based on periodic task timings (t) in seconds, momentary rates (f) in cycles per second were calculated: *f*_*n*_ = 1/(*t*_*n*_−*t*_*n*−1_), where t_n_ is the timing of an individual repetition, t_n–1_ is the timing of the previous repetition, and f_n_ is the momentary rate at t_n_. An array of momentary rates (F_j_) was thus calculated for each periodic task. Also, every repetition of one periodic task was paired exclusively with a repetition from the other task, such that each one in a pair of corresponding repetitions was temporally the most proximal counterpart to the other. For example, in a 15-s dual task trial of tapping and ticking by a subject in the study (see Figure 3), the subject produced the taps and the ‘tick’s in approximately a 2:1 ratio. There were 17 ticks against 33 taps in the trial. Each of the ticks, from 1 to 17 in ascending order, was paired exclusively with the corresponding tap from the array comprised of the 17 odd-numbered taps, from 1 to 33 in ascending order. For each trial, after such pairings, a two-way difference in timing between the counterparts in each pair was calculated, yielding two arrays of relative asynchronies, one for each periodic task (td_j_); the corresponding values of the two arrays were identical in magnitude but opposite in sign. Arrays of relative phase angles (θ_j_), one for each periodic task, were then calculated: θ_*j*_ = *td*_*j*_ × *F*_*j*_ × 2π. Applying the model as explained above, phase coherence (*r*) for each periodic task, relative to the other, was then calculated.

#### 2.4.5 Statistical analyses

For both experiments, phase coherence of each periodic task, relative to the other, was analyzed with a 2 × 2 repeated measures ANOVA: (*task:* single, dual) × (*stage:* pre-load, post-load). Then, phase coherence of tapping, relative to walking and ticking, was analyzed across pre-load and post-load stages with 2 × 2 repeated measures ANOVAs: (*task:* single, dual) × (*co-task:* walking, ticking).

For Experiment 1, phase coherences of tapping and walking, relative to each other, were analyzed with a 2 × 2 repeated measures ANOVA: (*load condition:* expecting-load, enduring-load) × (*load level:* 1, 2), and two one-way repeated measures ANOVAs with four within-subject levels each: (*load condition:* pre-load dual task, expecting-load (level 1 and 2), post-load dual task), and (*load condition:* pre-load dual task, enduring-load (level 1 and 2), post-load dual task).

For Experiment 2, phase coherences of tapping and ticking, relative to each other, were analyzed with a 2 × 6 repeated measures ANOVA: (*load condition:* expecting-load, enduring-load) × (*load level:* 1 to 6), and two one-way repeated measures ANOVAs with eight within-subject levels each: (*load condition:* pre-load dual task, expecting-load (level 1 to 6), post-load dual task), and (*load condition:* pre-load dual task, enduring-load (level 1 to 6), post-load dual task).

Post hoc tests with Bonferroni correction as well as with no correction were conducted as required. All hypothesis tests used α = 0.05 for significance.

## 3 Results

### 3.1 Experiment 1—tapping and walking

#### 3.1.1 Phase coherence during single task vs. dual task - 2 × 2 repeated measures ANOVA

**FIGURE 4:**
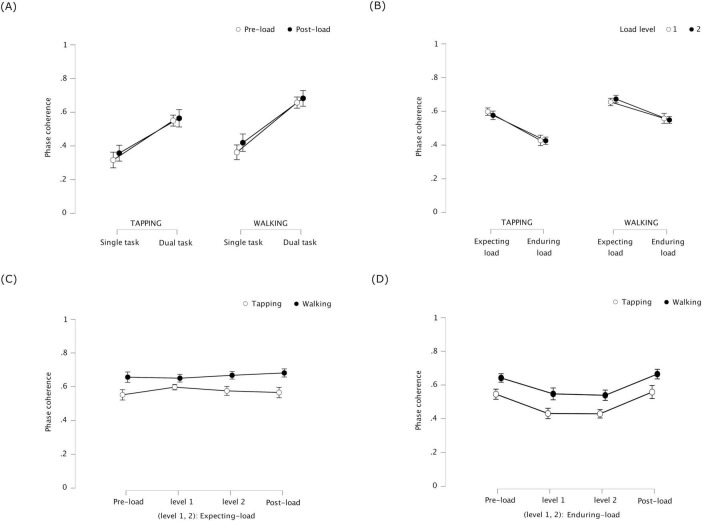
Experiment 1: phase coherence of tapping and walking representing coordination stability between the periodic tasks – (error bars indicate standard error) **(A)** Single task / dual task × pre-load stage / post-load stage – coordination stability increased between simultaneous tapping and walking (dual task) compared to separate (single task); the increase did not significantly differ between pre-load or post-load. **(B)** Expecting-load / enduring-load × load level (1 and 2) – coordination stability decreased in the tapping-walking dual task under cognitive load (enduring load) compared to Expecting load; the decrease was not significantly different across load levels 1 and 2 (counting backward in 3′s and 7′s, respectively). **(C)** Pre-load dual task / expecting-load (level 1 and 2) / post-load dual task – coordination stability was similar in the tapping-walking dual task between expecting cognitive load versus not (pre-load and post-load). **(D)** Pre-load dual task / enduring-load (level 1 and 2) / post-load dual task –coordination stability decreased in the tapping-walking dual task while enduring cognitive load versus not (pre-load and post-load).

Phase coherence was significantly higher during dual task than during single task, *F*(1, 23) = 11.331, *p* = 0.003, η^2^_p_ = 0.330 for tapping, and *F*(1, 23) = 19.690, *p* < 0.001, η^2^_p_ = 0.461 for walking (see Figure 4A). This indicates that tapping and walking were more coordinated when performed simultaneously than separately. Phase coherence did not significantly differ between pre-load and post-load stages, *F*(1, 23) = 0.712, *p* = 0.407, η^2^_p_ = 0.030 for tapping, and *F*(1, 23) = 1.514, *p* = 0.231, η^2^_p_ = 0.062 for walking. No significant interaction was found between task and stage, *F*(1, 23) = 0.246, *p* = 0.624, η^2^_p_ = 0.011 for tapping, and *F*(1, 23) = 0.305, *p* = 0.586, η^2^_p_ = 0.013 for walking.

#### 3.1.2 Phase coherence during expecting-load vs. enduring-load - 2 × 2 repeated measures ANOVA

Phase coherence was significantly lower while enduring load than while expecting load, *F*(1, 23) = 18.587, *p* < 0.001, η^2^_p_ = 0.447 for tapping, and *F*(1, 23) = 9.399, *p* = 0.005, η^2^_p_ = 0.290 for walking (see Figure 4B). This indicates that, coordination stability between tapping and walking during dual task decreased when participants concurrently counted backward compared to when they were only expecting to count backward. Phase coherence was not significantly different across load levels 1 and 2, *F*(1, 23) = 0.407, *p* = 0.530, η^2^_p_ = 0.017 for tapping, and *F*(1, 23) = 0.062, *p* = 0.805, η^2^_p_ = 0.003 for walking, indicating that counting backward by 3′s versus 7′s did not significantly differ in terms of how much they affected coordination stability. There was no significant interaction between load condition and load level, *F*(1, 23) = 0.558, *p* = 0.463, η^2^_p_ = 0.024 for tapping, and *F*(1, 23) = 1.228, *p* = 0.279, η^2^_p_ = 0.051 for walking.

#### 3.1.3 Phase coherence during pre-load, expecting-load and post-load: one-way repeated measures ANOVA with four within-subject levels

Phase coherence did not significantly differ between pre-load dual task, expecting load (level 1 and 2), and post-load dual task, *F*(3, 69) = 1.050, *p* = 0.377, η^2^_p_ = 0.044 for tapping, and *F*(3, 69) = 0.709, *p* = 0.550, η^2^_p_ = 0.030 for walking (see Figure 4C). This indicates that the effect of expecting load on coordination stability between tapping and walking was similar to that of no such expectation.

#### 3.1.4 Phase coherence during pre-load, enduring-load and post-load: one-way repeated measures ANOVA with four within-subject levels

Phase coherence was significantly different between pre-load dual task, enduring load (level 1 and 2), and post-load dual task, *F*(3, 69) = 7.465, *p* < 0.001, η^2^_p_ = 0.245 for tapping, and *F*(3, 69) = 7.36, *p* = 0.001, η^2^_p_ = 0.242 for walking (see Figure 4D). Post hoc tests with Bonferroni correction revealed that, (1) compared to pre-load dual task, phase coherence was significantly lower for tapping while enduring load level 1, *t*(23) = 3.084, *p* = 0.031, as well as level 2, *t*(23) = 3.870, *p* = 0.005; it was significantly lower for walking while enduring load level 2, *t*(23) = 3.416, *p* = 0.014, but not level 1, and (2) compared to post-load dual task, phase coherence was significantly lower for tapping while enduring load level 2, *t*(23) = 3.206, *p* = 0.024, but not level 1; it was significantly lower for walking while enduring load level 2, *t*(23) = 3.684, *p* = 0.007, but not level 1. Overall, except for tapping during pre-load dual task, concurrent counting backward in 3′s did not affect coordination stability between tapping and walking. On the other hand, counting backward in 7′s effected a significant decrease in coordination stability between tapping and walking across all conditions the cognitive task was performed concurrently.

### 3.2 Experiment 2—tapping and ticking

#### 3.2.1 Phase coherence during single task vs. dual task - 2 × 2 repeated measures ANOVA

**FIGURE 5:**
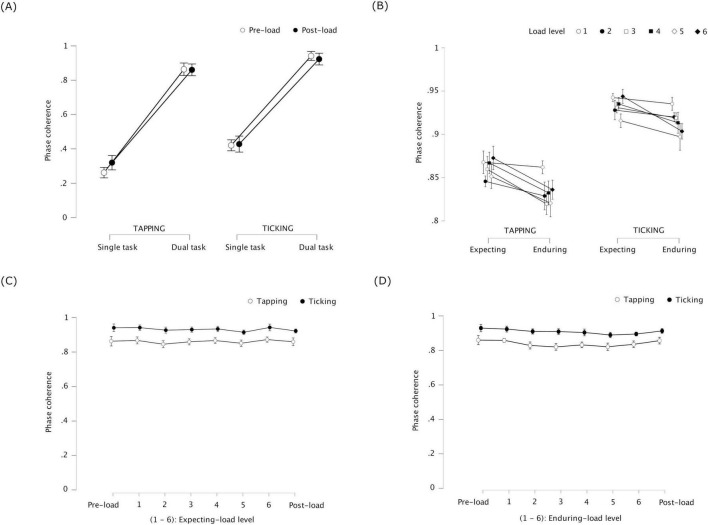
Experiment 2: phase coherence of tapping and ticking representing coordination stability between the periodic tasks – (error bars indicate standard error). **(A)** Single task / Dual task × pre-load stage / post-load stage –coordination stability increased between tapping and ticking when performed simultaneously (dual task) compared to separately (single task); coordination before cognitive load was introduced (pre-load) did not differ significantly from the same after load was removed (post-load). **(B)** Expecting-load / enduring-load × load level (1 to 6) – coordination stability decreased in the tapping-ticking dual task while enduring load compared to expecting load; the decrease did not significantly differ across load levels 1 to 6 (matching patterns of 4–9 blocks, respectively), except during level 3 when the coordination stability of ticking was significantly lower compared to level 1. **(C)** Pre-load dual task / expecting-load (level 1 to 6) / post-load dual task – coordination stability was similar in the tapping-ticking dual task between expecting cognitive load compared to not expecting load (pre-load and post-load). **(D)** Pre-load dual task / enduring-load (level 1 to 6) / post-load dual task – coordination stability was similar in the tapping-ticking dual task while enduring cognitive load compared to not (pre-load and post-load).

Phase coherence was significantly higher during dual task than during single task, *F*(1, 23) = 129.13, *p* < 0.001, η^2^_p_ = 0.849 for tapping, and *F*(1, 23) = 104.363, *p* < 0.001, η^2^_p_ = 0.819 for ticking (see Figure 5A). This indicates that tapping and ticking were more synchronous when performed simultaneously than separately. Phase coherence did not significantly differ between pre-load and post-load stages, *F*(1, 23) = 0.902, *p* = 0.352, η^2^_p_ = 0.038 for tapping, and *F*(1, 23) = 0.048, *p* = 0.829, η^2^_p_ = 0.002 for ticking. No significant interactions were found between task and stage, *F*(1, 23) = 1.782, *p* = 0.195, η^2^_p_ = 0.072 for tapping, and *F*(1, 23) = 0.255, *p* = 0.618, η^2^_p_ = 0.011 for ticking.

#### 3.2.2 Phase coherence during expecting-load vs. enduring-load - 2 × 6 repeated measures ANOVA

Phase coherence was significantly lower while enduring load than while expecting load, *F*(1, 23) = 7.966, *p* = 0.010, η^2^_p_ = 0.257 for tapping, and *F*(1, 23) = 4.820, *p* = 0.039, η^2^_p_ = 0.173 for ticking (see Figure 5B). This indicates that, when performing the periodic tapping and ticking tasks simultaneously, coordination stability decreased when participants were concurrently matching patterns compared to when they were only expecting to match patterns. Across load levels 1 to 6, phase coherence was not significantly different for tapping, *F*(5, 115) = 1.233, *p* = 0.298, η^2^_p_ = 0.051, but it was significantly different for ticking, *F*(5, 115) = 2.690, *p* = 0.024, η^2^_p_ = 0.105. Post hoc tests with Bonferroni correction revealed phase coherence of ticking to be significantly lower for load level 3 compared to level 1, *t*(23) = 4.445, *p* = 0.003. With no correction, compared to load level 1, phase coherence of ticking was significantly lower for level 3, *t*(23) = 4.445, *p* < 0.001, for level 4, *t*(23) = 2.186, *p* = 0.039, for level 5, *t*(23) = 2.501, *p* = 0.020, and for level 6, *t*(23) = 2.832, *p* = 0.009. No significant interaction was found between load condition and load level, *F*(5, 115) = 0.951, *p* = 0.451, η^2^_p_ = 0.040 for tapping, and *F*(5, 115) = 1.390, *p* = 0.233, η^2^_p_ = 0.057 for ticking.

#### 3.2.3 Phase coherence during pre-load, expecting-load and post-load: one-way repeated measures ANOVA with eight within-subject levels

Phase coherence did not significantly differ between pre-load dual task, expecting-load (levels 1 to 6), and post-load dual task conditions, *F*(7, 161) = 0.382, *p* = 0.912, η^2^_p_ = 0.016 for tapping, and *F*(7, 161) = 1.720, *p* = 0.108, η^2^_p_ = 0.069 for ticking (see Figure 5C). This indicates that the effect of expecting load on coordination stability between tapping and ticking was similar to that of no such expectation.

#### 3.2.4 Phase coherence during pre-load, enduring-load and post-load: one-way repeated measures ANOVA with eight within-subject levels

Phase coherence did not significantly differ between pre-load dual task, enduring-load (levels 1 to 6), and post-load dual task conditions, *F*(7, 161) = 1.410, *p* = 0.205, η^2^_p_ = 0.058 for tapping; it differed significantly for ticking, *F*(7, 161) = 2.162, *p* = 0.040, η^2^_p_ = 0.086 for ticking (see Figure 5D). However, *post hoc* comparisons with Bonferroni correction revealed no significant difference for ticking. This indicates that concurrent visual pattern-matching did not affect coordination stability between tapping and ticking.

#### 3.2.5 Phase coherence of tapping with walking and ticking – 2 × 2 repeated measures ANOVAs

Phase coherence of tapping, relative to walking and ticking, was significantly higher during dual task compared to single task, *F*(1, 23) = 102.496, *p* < 0.001, η^2^_p_ = 0.817 for pre-load stage, and *F*(1, 23) = 63.960, *p* < 0.001, η^2^_p_ = 0.736 for post-load stage. This indicates that tapping was more coordinated with walking and ticking when performed simultaneously than separately. Phase coherence of tapping was significantly higher with ticking compared to with walking, *F*(1, 23) = 8.265, *p* = 0.009, η^2^_p_ = 0.264 for pre-load stage, and *F*(1, 23) = 4.445, *p* = 0.046, η^2^_p_ = 0.162 for post-load stage. This indicates that tapping was more coordinated with ticking than with walking. There was a significant interaction between task condition (single task, dual task) and co-periodic task (walking, ticking), *F*(1, 23) = 23.044, *p* < 0.001, η^2^_p_ = 0.500 for pre-load stage, and *F*(1, 23) = 10.217, *p* = 0.004, η^2^_p_ = 0.308 for post-load stage. This indicates that tapping was more coordinated during dual task (compared to single task) with ticking than with walking.

## 4 Discussion

The current study investigated whether the stability of spontaneous intrapersonal coordination between periodic behaviors decreased more when walking was involved. In the dual task condition, at preferred rates, the stability of spontaneous coordination between finger-tapping and walking was significantly lower than that between finger-tapping and repetitive vocalization of the word “tick” (ticking). This finding is similar to that by Qi et al. (2019), where no evidence of coordination was found between tapping and walking, although spontaneous coordination occurred between tapping and foot movements; in that study, tapping was at an unrelated given rate. Therefore, at both preferred as well as given rates, spontaneous coordination of tapping is lower with walking than with other periodic tasks. This finding supports the categorization of walking as a more complex cognitive task compared to other periodic tasks (Sheridan and Hausdorff, 2007; Hausdorff et al., 2005), as the additional attentional load involved in walking could have resulted in lower coordination stability between tapping and walking.

**FIGURE 6:**
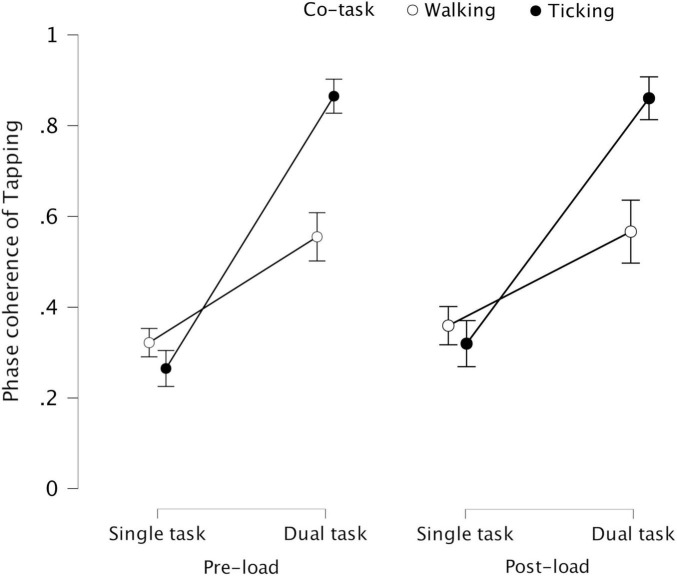
Phase coherence of tapping representing its coordination stability with walking and ticking in single task and dual task conditions across pre-load and post-load stages – (error bars indicate standard error) – The increase in coordination stability of tapping during dual task compared to single task was significantly greater with ticking than with walking, possibly due to higher attentional cost of walking.

In both experiments conducted in the current study, spontaneous intrapersonal coordination between the periodic tasks was significantly higher when the tasks were performed simultaneously than separately, in line with past findings. Although the direction of change in coordination stability across conditions was similar in both experiments, the effect sizes indicated that the magnitude of such change was different. In the dual task condition, the coordination stability increased less between tapping and walking than between tapping and ticking (see Figure 6). This could have been due to additional attentional cost of walking rendering tapping-walking coordination more difficult to achieve. Also, with additional load through concurrent cognitive task, the coordination stability decreased more between tapping and walking than between tapping and ticking. This could be due to backward counting being more efficient than visual pattern-matching in causing task interference: tapping-walking coordination decreased more with concurrent backward counting in 7′s than in 3′s, whereas tapping-ticking coordination was unaffected by the difficulty level of concurrent pattern-matching. Alternatively, this could again be due to additional attentional cost of walking causing tapping-walking coordination to be more susceptible to task interference, implying the possibility of cognitive overload.

Cognitive overload occurs when cognitive load imposed by processing demands exceeds the available resources, and this happens in three scenarios (Mayer and Moreno, 2003). Firstly, overload can be due to excessive demands in “essential processing” relevant to the core demands of the task; this is equivalent to Cognitive Load Theory’s “intrinsic cognitive load” that is imposed by the nature of the presented task, such as an arithmetic problem (Sweller et al., 1998; Sweller, 2011). In the current study, counting backward in 3′s and 7′s as well as matching visual patterns may have caused cognitive overload, either on their own or in conjunction with maintaining periodic tasks at a constant rate, which also may need cognitive resources. Secondly, demands in “incidental processing” irrelevant to the core task, on top of the essential processing demands, can cause overload; this is in line with Cognitive Load Theory’s “extraneous cognitive load” on top of “intrinsic cognitive load,” causing overload, where extraneous cognitive load is imposed by demands irrelevant to the core task, such as instructions that are hard to follow, or manner of task presentation, such as an illegible font in a reading comprehension task (Sweller et al., 1998; Sweller, 2011). In the current study, there were no obvious elements that fall into this category. Although participants needed to remember task instructions (e.g., to stay near to the microphone during ticking), these were not intentionally made to be difficult. Also, there were no coordination differences between the expecting-load condition and the pre-load or post-load stage, although they needed to remember additional task instructions while expecting load. Any extraneous load imposed by incidental processing demands are common in research, and seem reasonable enough not to be considered a cognitive overload risk. However, the possibility cannot be ruled out. Lastly, cognitive overload can be due to demands in “representational holdings” that refer to visual or auditory representations held in working memory (Mayer and Moreno, 2003). In the current study, the counting backward task required the participants to remember the current number in working memory (representational holding), until the next number was computed by applying the negative counter (essential processing); the visual pattern-matching task required them to remember the first pattern which was removed after a brief presentation (representational holding), until the second one was presented for comparison between the two patterns (essential processing). In both cognitive tasks, the combination of the two demands, posed by essential processing and representational holdings, could have caused cognitive overload, rendering the required resources for coordination unavailable.

Intrapersonal coordination may involve similar processes to interpersonal synchronization. One system related to interpersonal synchronization is the error monitoring / correction system under the predictive coding framework (Shamay-Tsoory et al., 2019; Gebauer et al., 2016; Koban et al., 2019). In particular, interpersonal synchronization minimizes “coding costs by reducing the mismatch between the representations of observed and own motor behavior” (Koban et al., 2019, p.1). Based on this postulate, high cognitive load should increase spontaneous interpersonal synchronization (2019) to relieve cognitive resources to support the cognitive task. The converse of this prediction is supported by previous findings: interpersonal synchronization imposed intentionally improves cognitive performance on problem-solving and memory tasks (Miles et al., 2017; Valdesolo et al., 2010; Von Zimmermann and Richardson, 2016; Woolhouse et al., 2016). In the current study, though, intrapersonal coordination stability decreased overall under additional cognitive load, suggesting that this prediction about interpersonal synchronization may not extend to intrapersonal coordination. However, it is important to consider that coordination stability between tapping and walking was significantly lower during enduring load compared to expecting load, but not compared to dual task with no such expectation. This suggests a possible increase in coordination stability during expecting load compared to dual task with no such expectation (see Figure 4C). Such an increase would be in line with the aforesaid postulate by Koban et al. (2019) in which case, our findings could have been due to cognitive overload. It would therefore be interesting to test cognitive loads that tax the limited resources but avoid overload.

Furthermore, under the predictive coding framework, error correction activates the reward system (Shamay-Tsoory et al., 2019) and triggers dopamine / oxytocin release (Gvirts and Perlmutter, 2020) that may, in turn, improve interpersonal synchrony by increasing the salience of social cues between interacting partners (Gvirts Probolovski and Dahan, 2021). This reasoning is supported by oxytocin improving interpersonal synchronization (Gebauer et al., 2016), and dopaminergic deficits impairing interpersonal synchronization, for example, as in Attention Deficit Hyperactivity Disorder (ADHD) (Problovski et al., 2021). Extending this to intrapersonal coordination, one could compare whether ADHD reduces intrapersonal coordination stability. In the current study, details of ADHD diagnosis were not collected from the participants, precluding any such comparison.

### 4.1 Limitations

The choice to use different cognitive tasks across the two experiments was due to the incompatibility of backward counting with repetitive vocalization. However, because of that, we could not meaningfully compare tapping-walking and tapping-vocalization in terms of coordination stability with concurrent cognitive tasks. Cognitive overload could have influenced the findings in the current study, masking any increase in spontaneous intrapersonal coordination under high cognitive load that is within the individual cognitive capacities of the participants. A tailored approach could have improved cognitive load manipulation, where load levels for each participant would be titrated to their individual ability. Also, given that spontaneity was the primary focus of investigation in the study, a balanced split of musicians and non-musicians would have allowed us to examine whether formal music training affected spontaneous intrapersonal coordination.

### 4.2 Future directions

It would be interesting to examine the effects of age and music training on spontaneous intrapersonal coordination. The ability to synchronize with external stimuli, key to interpersonal synchronization, is not affected by aging (Turgeon et al., 2011), but it is helped by music training (Repp, 2010; Scheurich et al., 2018). Whether these results on aging and music training apply to spontaneous intrapersonal coordination as well would be a logical inquiry to make. To address the cognitive overload issue, a follow-up using cognitive tasks with lower processing demands would be informative. To reduce the cognitive demands of a task, minimizing the need for representational holding by presenting the task information simultaneously instead of sequentially has been recommended (Mayer and Moreno, 2003). This can be applied in the visual pattern-matching task by presenting both patterns simultaneously instead of sequentially. Another possibility is to use pre-experiment individualized assessment of cognitive load capacity for each participant (Mayer and Moreno, 2003; Elliott et al., 2009). Also, individual differences in cognitive load capacity could be used to predict individual differences in spontaneous intrapersonal coordination. Extending this to interpersonal interactions that involve individuals with a wide range of cognitive capacities, it would be interesting to test if individual cognitive capacities predict the level of interpersonal coordination. Given how intra- and interpersonal coordination may have the same underlying sensorimotor control mechanisms at the sub-movement level (Nazzaro et al., 2023), it would be reasonable to evaluate if such a chain of predictions is viable. While it is reasonable to intuit intrapersonal coordination stability to transfer to interpersonal coordination, it is also important to consider findings that show that the strength of intrapersonal coupling interferes with what is potentially an aspect in interpersonal coordination: Learning of unfamiliar coordination patterns (Annand et al., 2020); although this interference is more in individuals than in dyads, and manageable with training, it is a factor that needs consideration. Overall, it will be interesting to see if and how intrapersonal coordination unfolds as a microcosm of its interpersonal counterpart.

## 5 Conclusion

Spontaneous intrapersonal coordination appears to increase between periodic behaviors when performed simultaneously compared to separately, and this increase is less pronounced between tapping and walking than between tapping and ticking. Also, additional cognitive load through a concurrent cognitive task decreases spontaneous intrapersonal coordination, and this decrease is more pronounced between tapping and walking than it is between tapping and ticking. Walking may be more cognitively demanding than ticking, thus more difficult to coordinate under additional cognitive load. Spontaneous intrapersonal coordination appears to be sensitive to the attentional costs of periodic behaviors and their coordination, thus may index cognitive capacity. Overall, the study demonstrates spontaneous intrapersonal coordination as a viable area of investigation into spontaneous coordination in general, and opens the door to further inquiry into how periodic behaviors interact within individuals.

## Data Availability

The raw data supporting the conclusions of this article will be made available by the authors, without undue reservation.

## Footnotes

1
https://audacityteam.org
